# Intermittent Moderate Energy Restriction Improves Weight Loss Efficiency in Diet-Induced Obese Mice

**DOI:** 10.1371/journal.pone.0145157

**Published:** 2016-01-19

**Authors:** Radhika V. Seimon, Yan-Chuan Shi, Katy Slack, Kailun Lee, Hamish A. Fernando, Amy D. Nguyen, Lei Zhang, Shu Lin, Ronaldo F. Enriquez, Jackie Lau, Herbert Herzog, Amanda Sainsbury

**Affiliations:** 1 The Boden Institute of Obesity, Nutrition, Exercise & Eating Disorders, Sydney Medical School, Charles Perkins Centre, The University of Sydney, Camperdown, Australia; 2 Neuroscience Division, Garvan Institute of Medical Research, Darlinghurst, Australia; 3 Bone Biology Division, Garvan Institute of Medical Research, St Vincent's Hospital, Sydney, NSW, Australia; Institut d'Investigacions Biomèdiques August Pi i Sunyer, SPAIN

## Abstract

**Background:**

Intermittent severe energy restriction is popular for weight management. To investigate whether intermittent moderate energy restriction may improve this approach by enhancing weight loss efficiency, we conducted a study in mice, where energy intake can be controlled.

**Methods:**

Male C57/Bl6 mice that had been rendered obese by an *ad libitum* diet high in fat and sugar for 22 weeks were then fed one of two energy-restricted normal chow diets for a 12-week weight loss phase. The continuous diet (CD) provided 82% of the energy intake of age-matched *ad libitum* chow-fed controls. The intermittent diet (ID) provided cycles of 82% of control intake for 5–6 consecutive days, and *ad libitum* intake for 1–3 days. Weight loss efficiency during this phase was calculated as (total weight change) ÷ [(total energy intake of mice on CD or ID)–(total average energy intake of controls)]. Subsets of mice then underwent a 3-week weight regain phase involving *ad libitum* re-feeding.

**Results:**

Mice on the ID showed transient hyperphagia relative to controls during each 1–3-day *ad libitum* feeding period, and overall ate significantly more than CD mice (91.1±1.0 versus 82.2±0.5% of control intake respectively, *n* = 10, P<0.05). There were no significant differences between CD and ID groups at the end of the weight loss or weight regain phases with respect to body weight, fat mass, circulating glucose or insulin concentrations, or the insulin resistance index. Weight loss efficiency was significantly greater with ID than with CD (0.042±0.007 versus 0.018±0.001 g/kJ, *n* = 10, P<0.01). Mice on the CD exhibited significantly greater hypothalamic mRNA expression of proopiomelanocortin (POMC) relative to ID and control mice, with no differences in neuropeptide Y or agouti-related peptide mRNA expression between energy-restricted groups.

**Conclusion:**

Intermittent moderate energy restriction may offer an advantage over continuous moderate energy restriction, because it induces significantly greater weight loss relative to energy deficit in mice.

## Introduction

Weight loss by lifestyle intervention is almost twice as effective as anti-diabetic drugs (metformin) for preventing the progression to overt diabetes in people with overweight or obesity [1]. Weight loss therefore offers a simple and powerful strategy for preventing not only type 2 diabetes but also other diseases. However, adhering to the long-term energy restriction that is required to achieve lasting, clinically significant weight loss is notoriously difficult. Improved weight loss strategies are urgently needed.

Studies in humans and animals increasingly suggest that intermittent energy restriction is on par with, or even superior to, continuous energy restriction with regards to various health benefits. These include improved glucose homeostasis [2–9], improved cardiovascular and cerebrovascular health [9–11], potentially reduced tumour initiation and growth [12,13], and delayed age-related neurodegeneration and cognitive impairments [14]. Against this background, weight reducing diets involving intermittent energy restriction have recently gained popularity amongst health professionals and members of the public alike [15,16]. Such eating patterns involve restricting energy intake by varying degrees for a pre-defined period of time, and eating *ad libitum* (i.e. to satisfy appetite)–or at least more than during the energy-restricted period—at all other times. The most common form of intermittent energy restriction is ‘intermittent fasting’, where energy intake is severely restricted to ~0–50% of energy requirements for short periods (typically 1 to 7 consecutive or non-consecutive days per week) [17]. During periods of greater energy intake, there may or may not be restrictions placed on the types and amounts of foods and beverages consumed [17].

Recent reviews of clinical trials in individuals with overweight or obesity suggest that intermittent severe energy restriction results in *equivalent*–albeit not superior—weight or fat loss and improvements in cardiovascular disease risk factors and fasting circulating insulin levels and insulin sensitivity (as assessed by homeostatic model assessment for insulin resistance, HOMA-IR) relative to moderate continuous energy restriction [17–20]. As such, intermittent severe energy restriction is increasingly recognised as a viable and equivalent option for weight loss [17].

One potential limitation of intermittent severe energy restriction is that it may not produce optimally efficient weight loss, because severe energy restriction as defined above has been shown to result in less weight loss per unit of energy restriction than more moderate energy restriction in adult humans with obesity [21–23]. We hypothesized that intermittent *moderate* energy restriction would improve the efficiency of weight loss relative to continuous moderate energy restriction. We define moderate energy restriction as a diet providing ~70–80% of energy requirements, as in human weight loss interventions that commonly restrict energy intake by ~2,000 kJ/day. Our rationale was that while energy restriction—notably severe energy restriction—is known to induce adaptive responses such as reduced energy expenditure that inhibit further weight loss and promote weight regain [24,25], some of these effects were reported to be normalized or attenuated by a period of *ad libitum* food intake or weight maintenance [26–31]. Thus, providing ‘metabolic rest periods’ via *ad libitum* feeding and increased food intake would be hypothesized to attenuate the energy-conserving effects of energy restriction, thereby improving weight loss efficiency (weight loss per unit energy restriction), potentially providing a more effective angle for human obesity treatment. We tested this hypothesis in mice, where manipulating energy intake and determining actual intake are relatively straightforward, unlike in humans, where adherence to specific prescriptions of energy intake and determining actual intake are challenging [32].

## Materials and Methods

### Ethics, animals, experimental design, calculation of weight loss efficiency, tissue collection and body composition assessment

All research and animal care procedures were approved by the Garvan Institute/St Vincent’s Hospital Animal Ethics Committee (approved protocol #04/27) and were in agreement with the Australian Code of Practice for the Care and Use of Animals for Scientific Purposes.

**Fig 1:**
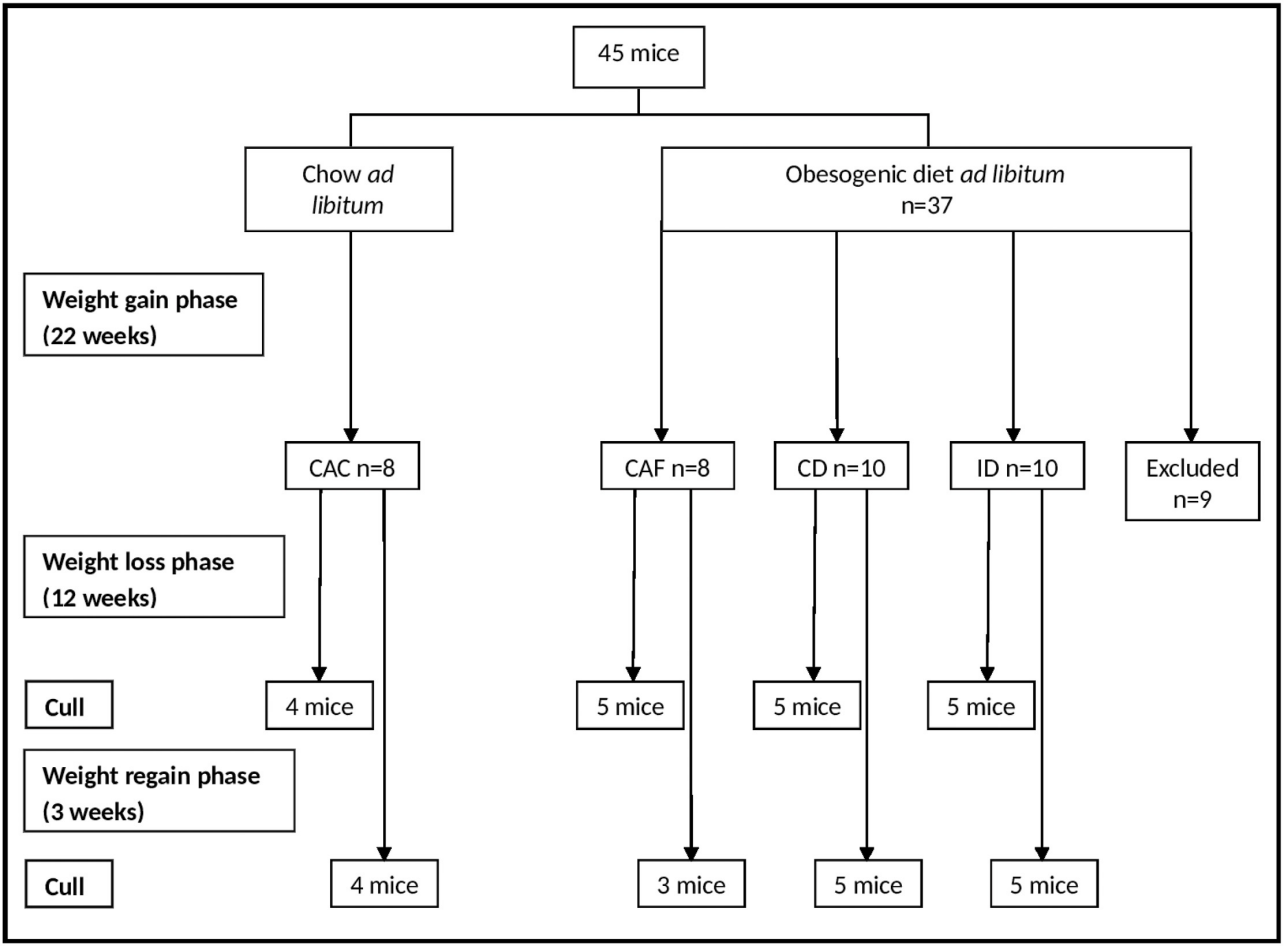
Study flow chart. CAC, continuous *ad libitum* chow; CAF, continuous *ad libitum* high fat; CD, continuous diet; ID, intermittent diet.

45 male C57/Bl6 mice at 3 weeks of age were purchased from the Animal Resources Centre (Canning Vale, Western Australia, Australia). Mice were housed under conditions of controlled temperature (22°C) and illumination (12-hour light-dark cycle, lights on at 7:00 hours). At 4 weeks of age, mice were housed in groups of 4–5 mice per cage and were allowed to feed *ad libitum* for 22 weeks on one of two diets as shown in Fig 1; normal chow (8 mice) or a high fat, high sugar (obesogenic) diet (37 mice). This period is referred to as the “weight gain phase”. The normal chow provided 8% of energy from fat, 21% of energy from protein, 71% of energy from carbohydrates, and 10.9 kJ/g (Gordon’s Specialty Stock Feeds, Yanderra, New South Wales, Australia). The high fat diet was normal chow supplemented with fat and sucrose. It provided 46% of energy from fat, 21% of energy from protein, 33% of energy from carbohydrates and 19.7 kJ/g. The diet was based on the composition of rodent diet (Catalog number: D12451; Research Diets, New Brunswick, NJ, USA), with the exception that safflower oil and copha were used in place of soybean oil and lard. All data for food intake are presented in units of energy intake (kJ/day) rather than weight consumed, due to the different energy densities of normal chow and the high fat diet.

During the weight gain phase, body weight and energy intake were determined approximately once a week at a similar time of day. Energy intake (kJ/day) was calculated as [[(weight of food placed in hopper)–(weight of food remaining in hopper)] x (energy density of food in kJ/g)] ÷ [(number of mice per cage) x (number of days)]. Following the weight gain phase, 9 mice that had not gained significant amounts of body weight despite 22 weeks on the high fat diet were excluded from the study (Fig 1). The body weight of these mice (n = 9; 29.2±0.6 g) was not significantly different from that of the age-matched chow-fed mice (n = 8; 28.2±0.6 g). This finding is in keeping with the observation that C57/Bl6 mice show variable responses to a high fat diet, with some individual animals being resistant to diet-induced weight gain and metabolic perturbations [33,34]. The remaining mice were divided into 4 experimental groups for a 12-week (85-day) “weight loss phase” as shown in Fig 1. Two control groups, namely the “continuous *ad libitum* chow” (CAC, n = 8) and “continuous *ad libitum* high fat diet” (CAF, n = 8), had *ad libitum* access to normal chow or the high fat diet, respectively, for this and all other phases of the experiment. The remaining obese, high fat fed mice were transferred from group housing to individual housing and were randomly allocated to either the “continuous diet” (n = 10) or the “intermittent diet” (n = 10). Mice in these two groups were allowed to recover normal food intake levels prior to commencing experimentally imposed energy restriction. Mice on the continuous diet and intermittent diet received normal chow for the weight loss phase. Mice on the continuous diet were given weighed portions of normal chow that were equivalent to 82% of the *ad libitum* chow intake of age-matched mice in the CAC group, mimicking the moderate energy restriction that is frequently prescribed for the dietary management of overweight and obesity in humans. Mice on the intermittent diet were alternated between 5- to 6-day periods (average 5.6 days) of restricted chow intake, at a level equivalent to that of mice in the continuous diet group, followed by 1- to 3- day periods (average 2.1 days), where they were allowed to eat normal chow *ad libitum*. Actual energy intake was determined daily in these individually housed mice as described above.

Weight loss efficiency from the start to the end of the weight loss phase was calculated as (total weight change for each mouse on the continuous or intermittent diet) ÷ (total energy deficit), where total energy deficit is (total energy intake of each mouse on the continuous or intermittent diet)–(total average energy intake of CAC controls). It is expressed in units of g/kJ (i.e. grams lost per kJ of energy restriction relative to the energy intake of age-matched control mice during the 12-week weight loss phase). This calculation is based on a deficit-efficiency factor of (body energy loss) ÷ (dietary energy deficit) that was previously developed to quantify the effectiveness of weight-reduction interventions [23].

At the end of the weight loss phase, 4 mice from the CAC group, 5 mice from the CAF, and 5 mice from each of the continuous diet and intermittent diet groups were fasted from 8:00 hours and then culled by cervical dislocation between 11:00 and 14:00 hours. Trunk blood was then collected and serum was subsequently collected and frozen at -80°C until analysis as described below. Brains were isolated and frozen hypothalamus side up, on an aluminium plate on dry ice. They were then stored at -80°C until subsequent analysis as described below. The carcasses were scanned (with the head excluded and the tail included) using dual energy X-ray absorptiometry (Lunar PIXImus2 mouse densitometer; GE Healthcare, Waukesha, WI, USA) to determine lean (g) and fat (% and g) mass. The following white adipose tissue (WAT) depots and other tissues were then dissected and weighed; the inguinal (WATi), epididymal (WATe), retroperitoneal (WATr) and mesenteric (WATm) WAT depots, the interscapular brown adipose tissue (BAT) depot, as well as the pancreas, liver, heart, kidney, spleen, testes and the seminal vesicles.

All remaining mice then proceeded to a 3-week (22-day) “weight regain phase”, where mice from the energy-restricted groups were given *ad libitum* access to normal chow for the first 10 days and to the high fat diet for the ensuing 12 days. Following the weight regain phase, mice were culled and the carcasses and tissues were collected as described above.

### Determination of fasting serum glucose, insulin and insulin resistance

Serum glucose and insulin concentrations were measured using a glucose oxidase kit from Trace Scientific (Clayton, Victoria, Australia) and a radioimmunoassay kit from Millipore Corporation (Billerica, MA, USA), respectively. From these, an index of insulin resistance was estimated using the formula for the homeostatic model assessment for insulin resistance (HOMA-IR), which is (fasting insulin [mU/L] x fasting glucose [mmol/L])/22.5 [35].

### Real time PCR

To determine the hypothalamic mRNA expression levels of neuropeptide Y (NPY), agouti-related peptide (AgRP) and proopiomelanocortin (POMC), the hypothalamic region of frozen brains was dissected out and total RNA was isolated using Trizol Reagent (Sigma, St Louis, MO, USA) following the manufacturer’s protocol. The quality and concentration of total RNA was measured with a spectrophotometer (Nanodrop 1000, NanoDrop Technologies, LLC, USA). 1 μg of total RNA was reverse transcribed into cDNA using the Superscript III First-Strand Synthesis System (Invitrogen, Mount Waverley, Victoria, Australia). Quantitative real-time PCR using primers for NPY, AgRP or POMC was carried out on a Light-Cycler^®^ 480 Real-Time PCR system (Roche Applied Science, Germany) using SensiMix^™^ Probe (Bioline Australia Pty Ltd, Alexandria, New South Wales, Australia) following the manufacturer’s instructions. Expression of the ribosomal protein L19 (RPL19) was used to normalize mRNA expression levels of genes of interest. Primers for NPY, AgRP and POMC were as listed below:

NPY: Forward: GAAAGCACAGAAAACGCCCCCAG, Reverse: AAATGGGGCGGAGTCCAGCCTA; AgRP: Forward: TTTGTCCTCTGAAGCTGTATGC, Reverse: GCATGAGGTGCCTCCCTA; POMC: Forward: AGTGCCAGGACCTCACCA, Reverse: CAGCGAGAGGTCGAGTTTG.

### Statistical analysis

Data were analyzed using SPSS version 18 (SPSS Inc, Chicago, Illinois, USA). Repeated-measures analysis of variance (ANOVA) was used to evaluate energy intake, body weight and WAT depot weights, with time or depot type as the within-subject factor, and group (CAC, CAF, continuous diet and intermittent diet) as the between-subject factor. Area under the curves (AUCs) for energy intake and body weight was calculated using the trapezoidal rule. One-way ANOVA was used to analyze AUCs for energy intake and body weight, as well as weight loss efficiency, body composition as determined by dual energy X-ray absorptiometry, the weight of dissected tissues (besides WAT depots), serum glucose and insulin concentrations, the insulin resistance index as well as hypothalamic NPY, AgRP and POMC mRNA expression levels, with group as a factor. *Post-hoc* comparisons, adjusted for multiple comparisons by Bonferroni's correction, were performed where ANOVA revealed significant effects. Statistical significance was accepted at P<0.05. Data are presented as means ± SEM.

## Results

### Energy intake was greater on the intermittent than on the continuous diet

**Fig 2:**
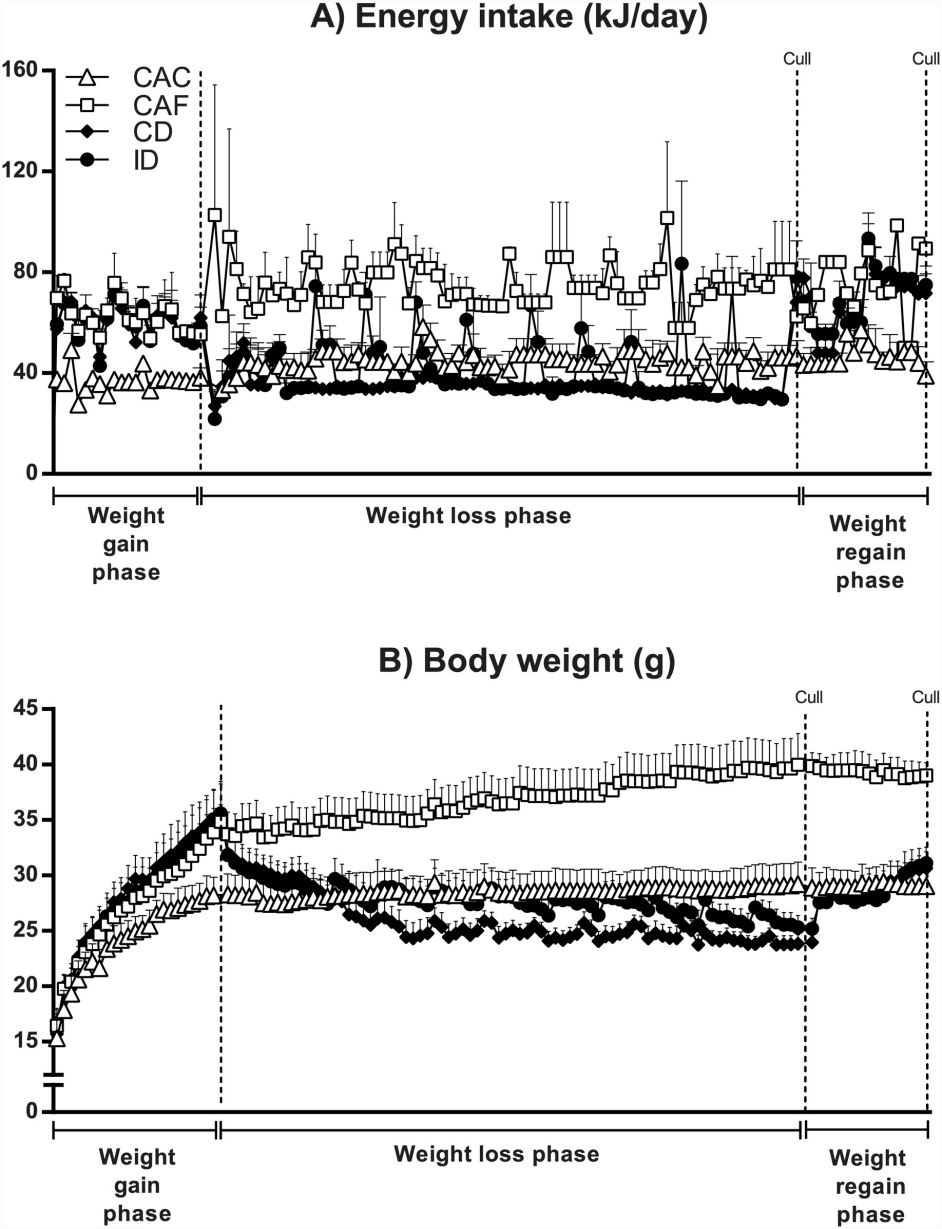
Energy intake (A) and body weight (B) during the weight gain, loss and regain phases. CAC, continuous *ad libitum* chow; CAF, continuous *ad libitum* high fat diet; CD, continuous diet; ID, intermittent diet. Data are means ± SEM of 3–10 mice per group as shown in Fig 1.

The daily energy intake of all 4 groups of mice throughout the 3 phases of the experiment (weight gain, weight loss and weight regain) are shown in Fig 2A, and the area under the curves (AUCs) for each group during each phase are shown in Table 1. As expected, mice in the CAF group consumed significantly more energy per day than mice in the CAC group throughout all 3 phases of the experiment. During the weight gain phase, mice in the continuous and intermittent diet groups, which also had *ad libitum* access to the high fat diet at this time, consumed a similarly elevated number of kJ per day relative to those in the CAF group.

**Table 1:** Energy intake, body weight and weight loss efficiency during the weight gain, loss or regain phases of the experiment.

	CAC	CAF	CD	ID
***AUC energy intake (kJ*.*day)***				
Weight gain phase	35.0±0.1	59.3±0.9*	57.5±0.9*	57.3±0.8*
Weight loss phase	42.2±1.1	68.1±1.9*	34.6±0.2*§	38.6±0.6§#
Weight regain phase: total	44.2±2.7	70.5±0**	63.6±1.1*	66.3±1.1*
Weight regain phase: chow	-	-	53.3±1.0	55.4±1.3
Weight regain phase: fat	-	-	66.8±1.7	69.8±1.0
***AUC Body weight (g*.*day)***				
Weight gain phase	23.2±0.5	26.0±0.4*	27.1±0.6*	26.6±0.6*
Weight loss phase	28.2±0.6	36.5±0.8***	25.5±0.3**§§§	27.4±0.4§§§
Weight regain phase: total	27.4±0.7	37.0±0.8***	27.2±0.6§§§	26.9±0.3§§§
Weight regain phase: chow	-	-	25.4±0.4	24.9±0.3
Weight regain phase: fat	-	-	25.7±0.5	25.7±0.2
***Body weight (g)***				
Start of weight gain phase	15.3±0.8	16.5±0.6	15.7±0.5	16.2±0.5
Start of weight loss phase	28.2±0.6	33.9±0.8***	35.0±0.8***	35.1±0.8***
Start of weight regain phase	29.2±0.7	40.0±1***	23.8±0.3***§§§	25.3±0.3***§§§
End of weight regain phase	29.1±0.7	39±0.7*	30.1±0.9§	31.1±0.5§
***Weight loss efficiency (g/kJ)***				
Weight loss phase	-	-	0.018±0.001	0.042±0.007##

CAC, continuous *ad libitum* chow; CAF, continuous *ad libitum* high fat diet; CD, continuous diet; ID, intermittent diet; AUC, area under the curve. Data are means ± SEM of the number of mice shown in Fig 1.

* P<0.05,

** P<0.01,

*** P<0.001 versus CAC.

^§^ P<0.05,

^§§^ P<0.01,

^§§§^ P<0.001 versus CAF.

^#^ P<0.05,

^##^ P<0.01 versus CD.

At the start of the weight loss phase there was a spontaneous drop in energy intake in mice in the continuous and intermittent diet groups, corresponding to their transfer from group housing to individual housing and their switch from the high fat diet to normal chow, but energy intake recovered to values indistinguishable from that of mice in the CAC group within a week (Fig 2A). For the remainder of the weight loss phase, the 24-hour energy intake of mice on the continuous diet was experimentally restricted to an overall average of 82% of that of mice in the CAC group, as shown in Fig 2A and Table 1. This level of energy intake was significantly less than that of CAC mice (Table 1). Mice on the intermittent diet did not eat significantly less overall than those in the CAC group during the weight loss phase (Table 1), despite the fact that on most days their energy intake was also experimentally restricted to an overall average of 82% of that of mice in the CAC group. This lack of overall difference in energy intake compared to CAC controls despite periods of energy restriction is because when mice on the intermittent diet were given *ad libitum* access to normal chow, they ate markedly more than mice in the CAC or continuous diet groups, as shown in Fig 2A. The average energy intake of these mice on the first and—when available—second and third day of *ad libitum* food intake was 139±9%, 105±3%, and 101±6% that of control mice in the CAC group. The result of these intermittent increases in energy intake is that mice on the intermittent diet consumed 11.5% more energy (P<0.05) than mice on the continuous diet over the entire weight loss phase (Table 1).

During the weight regain phase, when mice in both the continuous diet and intermittent diet groups were given *ad libitum* access to normal chow for the first 10 days and to the high fat diet for the ensuing 12 days, both groups showed similar increases in energy intake, with no significant difference between the two groups (Fig 2A, Table 1).

### No difference in final body weight between mice on the continuous or intermittent diet

There was no significant difference among the four groups of mice with respect to body weight at the start of the experiment at 4 weeks of age (Fig 2B, Table 1). As seen in Fig 2B and Table 1, mice in the CAF group had greater body weights than mice in the CAC group throughout all 3 phases of the experiment. During the weight gain phase, mice in the continuous diet and intermittent diet groups, which also had *ad libitum* access to the high fat diet at this time, had a similarly elevated body weight to those in the CAF group (Fig 2B, Table 1).

At the start of the weight loss phase there was a drop in body weight in mice in the continuous diet and intermittent diet groups, corresponding to their transient spontaneous hypohagia (Fig 2). Mice on the continuous diet had a lower body weight than those on the intermittent diet during the first part of the weight loss phase, but by the end of the weight loss phase (i.e. at the start of the weight regain phase) there was no significant difference between these two groups with respect to body weight (Fig 2B, Table 1), and no difference overall as assessed by ANOVA or AUC (Table 1). It is noteworthy that the body weight of both groups of energy-restricted mice was significantly lower than that of mice in the CAC group by the end of the weight loss phase (i.e. by the start of the weight regain phase) (Fig 2B, Table 1), indicating that their weight was lower than their defended body weight (‘set point’).

During the weight regain phase, mice in the continuous diet and intermittent diet groups showed similar body weights, with no significant difference between the two groups, and no significant difference from the body weight of mice in the CAC group in this timeframe (Fig 2B, Table 1).

### Weight loss efficiency was significantly greater on the intermittent than on the continuous diet

During the weight loss phase, there was a significant, 2.3-fold greater weight loss efficiency (total weight loss ÷ total energy deficit) for mice on the intermittent diet compared to those on the continuous diet (Table 1). This is because whereas both groups of energy-restricted mice exhibited similar total weight loss (Fig 2B, Table 1), the total energy deficit of mice on the intermittent diet during the 12-week weight loss phase (304.5±47.7 kJ) was less than half that of mice on the continuous diet (646.9±16.8 kJ). This means that for every kJ of deficit in energy intake relative to the energy intake of age-matched control mice, mice on the intermittent diet lost 2.3 times as much weight as mice on the continuous diet.

### No difference in body composition between mice on the continuous or intermittent diet

**Fig 3:**
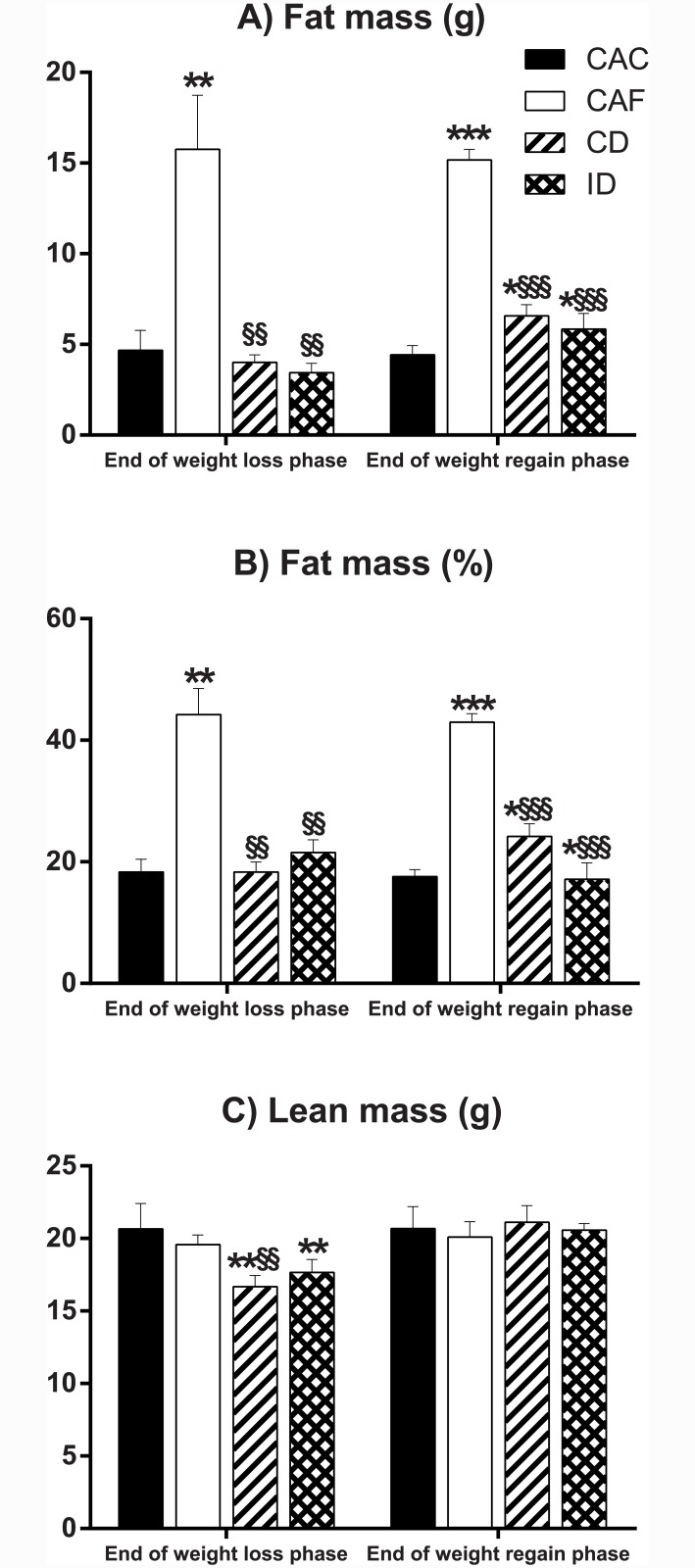
Body composition as determined by dual energy X-ray absorptiometry. (A) Fat mass (g), (B) fat mass (% of body weight) and (C) lean mass (g) at the end of the weight loss and weight regain phases. CAC, continuous *ad libitum* chow; CAF, continuous *ad libitum* high fat diet; CD, continuous diet; ID, intermittent diet. Data are means ± SEM of 3–10 mice per group as shown in Fig 1. * P<0.05, ** P<0.01, *** P<0.001 versus CAC. §§ P<0.01, §§§ P<0.001 versus CAF.

**Fig 4:**
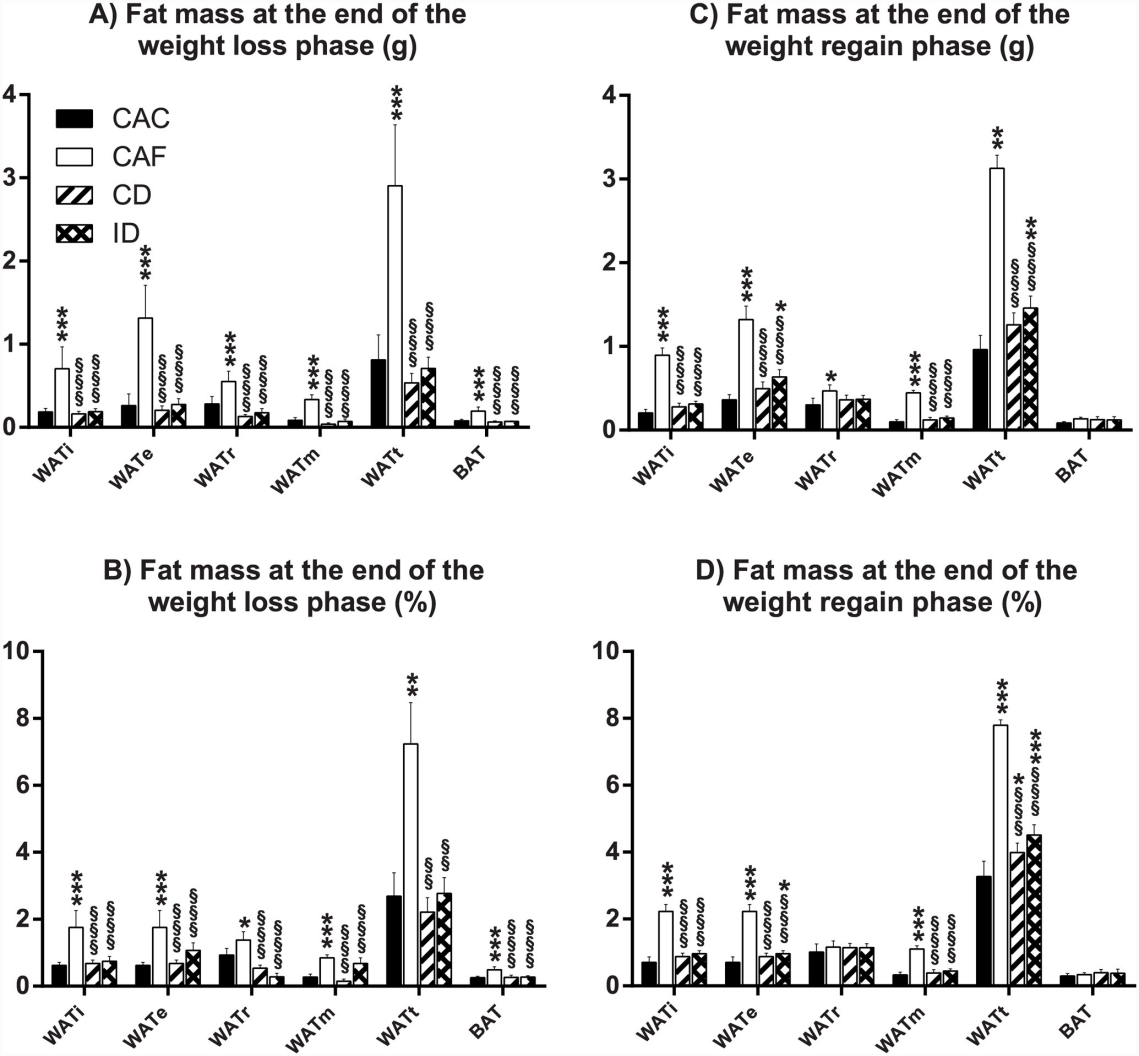
Body composition as determined by adipose tissue dissection. White adipose tissue (WAT) and brown adipose tissue (BAT) depot masses are presented in absolute weights (g) and as a percent of body weight (%) at the end of the weight loss (A and B) and weight regain (C and D) phases. CAC, continuous *ad libitum* chow; CAF, continuous *ad libitum* high fat diet; CD, continuous diet; ID, intermittent diet; WATi, inguinal WAT; WATe, epididymal WAT; WATr, retroperitoneal WAT; WATm, mesenteric WAT; WATt, sum of the above 4 WAT depot masses. Data are means ± SEM of 3–10 mice per group as shown in Fig 1. * P<0.05, ** P<0.01, *** P<0.001 versus CAC. §§§ P<0.001 versus CAF.

In keeping with obesity induced by *ad libitum* access to the high fat diet, mice in the CAF group exhibited significantly higher fat masses (both in absolute weight and as a percent of body weight)–as determined by dual energy X-ray absorptiometry (Fig 3A and 3B) as well as by adipose tissue depot dissection (Fig 4A–4D)—compared to mice in every other group. At the end of the weight loss phase, fat masses in the previously high fat-fed mice from the continuous and intermittent diet groups were significantly less than that of mice in the CAF group, with no significant difference between these two groups or the CAC group (Fig 3A and 3B, Fig 4A and 4B). At the end of the weight regain phase, fat masses of mice in the continuous and intermittent diet groups remained statistically indistinguishable from each other, albeit both groups exhibited significantly greater total fat mass as determined by dual energy X-ray absorptiometry (Fig 3A and 3B) but not necessarily by dissection of individual fat depots (Fig 4C and 4D) than mice in the CAC group at this time point.

As seen from data on lean mass measured at the end of the weight loss phase (Fig 3C), weight loss produced significant reductions in lean mass in mice on the continuous and intermittent diets relative to corresponding values from CAF and / or CAC groups, in keeping with a known effect of weight loss to reduce fat free mass [36]. However, there was no significant difference in lean mass between mice on either of the two energy-restricted diets at this time point (Fig 3C). At the end of the weight regain phase, there were no significant differences in lean mass amongst any of the groups (Fig 3C).

### The continuous but not the intermittent diet reduced the weight of male reproductive organs

Following the weight loss phase, but not the weight regain phase, there was a significant reduction in relative weight (as a percent of body weight) of the seminal vesicles of mice on the continuous but not on the intermittent diet (Table 2). There were no significant differences in testes weight among groups (Table 2). At the end of the weight loss and weight regain phases, there were no differences amongst groups with respect to absolute (g) or relative (%) weight of the pancreas, liver, heart, kidney or spleen (data not shown).

**Table 2:** Weight (g and as a % of body weight) of seminal vesicles and testes, and serum glucose and insulin levels, as well as insulin resistance index, at the end of the weight loss and weight regain phases of the experiment.

	CAC	CAF	CD	ID
***End of weight loss phase***				
*Seminal vesicles*				
Weight (g)	0.21±0.02	0.22±0.01*	0.11±0.01***§§	0.16±0.01***§§#
Weight (%)	0.69±0.05	0.56±0.04	0.47±0.02**	0.64±0.04#
*Testes*				
Weight (g)	0.110±0.004	0.102±0.006	0.082±0.015	0.100±0.003
Weight (%)	0.36±0.02	0.26±0.02	0.33±0.06	0.38±0.01
*Glucose / insulin*				
Glucose (mM)	6.8 ± 0.8	8.2 ± 0.8	4.6 ± 0.3§§	4.3 ± 0.3§§
Insulin (pM)	137 ± 18.7	235 ± 22.4*	59.3 ± 6.3§§§	84.3 ± 23.2§§§
Insulin resistance index	19.9 ± 0.7	29.8 ± 3.7	12.8 ± 1.1§	19.0 ± 4.8
***End of weight regain phase***				
*Seminal vesicles*				
Weight (g)	0.24±0.02	0.23±0.01	0.23±0.01	0.24±0.01
Weight (%)	0.80±0.05	0.57±0.02	0.74±0.05	0.74±0.02
*Testes*				
Weight (g)	0.113±0.018	0.103±0.026	0.120±0.010	0.102±0.004
Weight (%)	0.38±0.06	0.26±0.06	0.38±0.03	0.32±0.01
*Glucose / insulin*				
Glucose (mM)	7.0 ± 0.6	8.5 ± 0.4	8.3 ± 0.3	8.1 ± 1.0
Insulin (pM)	126 ± 10.4	239 ± 25.9*	248 ± 26.1**	209 ± 24
Insulin resistance index	18.0 ± 1.2	28.2 ± 2.5	30.2 ± 3.5*	26.0 ± 2.1

CAC, continuous *ad libitum* chow; CAF, continuous *ad libitum* high fat diet; CD, continuous diet; ID, intermittent diet. Data are means ± SEM of 3–10 mice per group as shown in Fig 1.

* P<0.05,

** P<0.01,

*** P<0.001 versus CAC.

^§§^ P<0.01,

^§§§^ P<0.001 versus CAF.

^#^ P<0.05 versus CD.

### No difference in serum glucose, insulin or the insulin resistance index between mice on the continuous or intermittent diet

In keeping with an euglycemic obesity syndrome, mice in the CAF group exhibited no significant change in serum glucose and significantly greater serum insulin concentrations compared to corresponding values in the CAC group at the end of both the weight loss and weight regain phases (Table 2). At the end of the weight loss phase, mice on the continuous and intermittent diets had significantly lower serum glucose and insulin concentrations than those in the CAF group, with no significant difference between the intermittent and continuous diet groups or from the CAC group (Table 2). Both the continuous diet and intermittent diet groups exhibited a lower insulin resistance index than that of the CAF group, significantly so for the continuous diet group (Table 2). At the end of the weight regain phase, serum insulin concentrations and the insulin resistance index were significantly greater in the continuous but not the intermittent diet group relative to CAC mice, but values were not significantly different from mice in the CAF group (Table 2). Again, there was no significant difference between the continuous diet and the intermittent diet group (Table 2).

### The continuous but not the intermittent diet increased hypothalamic expression of proopiomelanocortin

**Fig 5:**
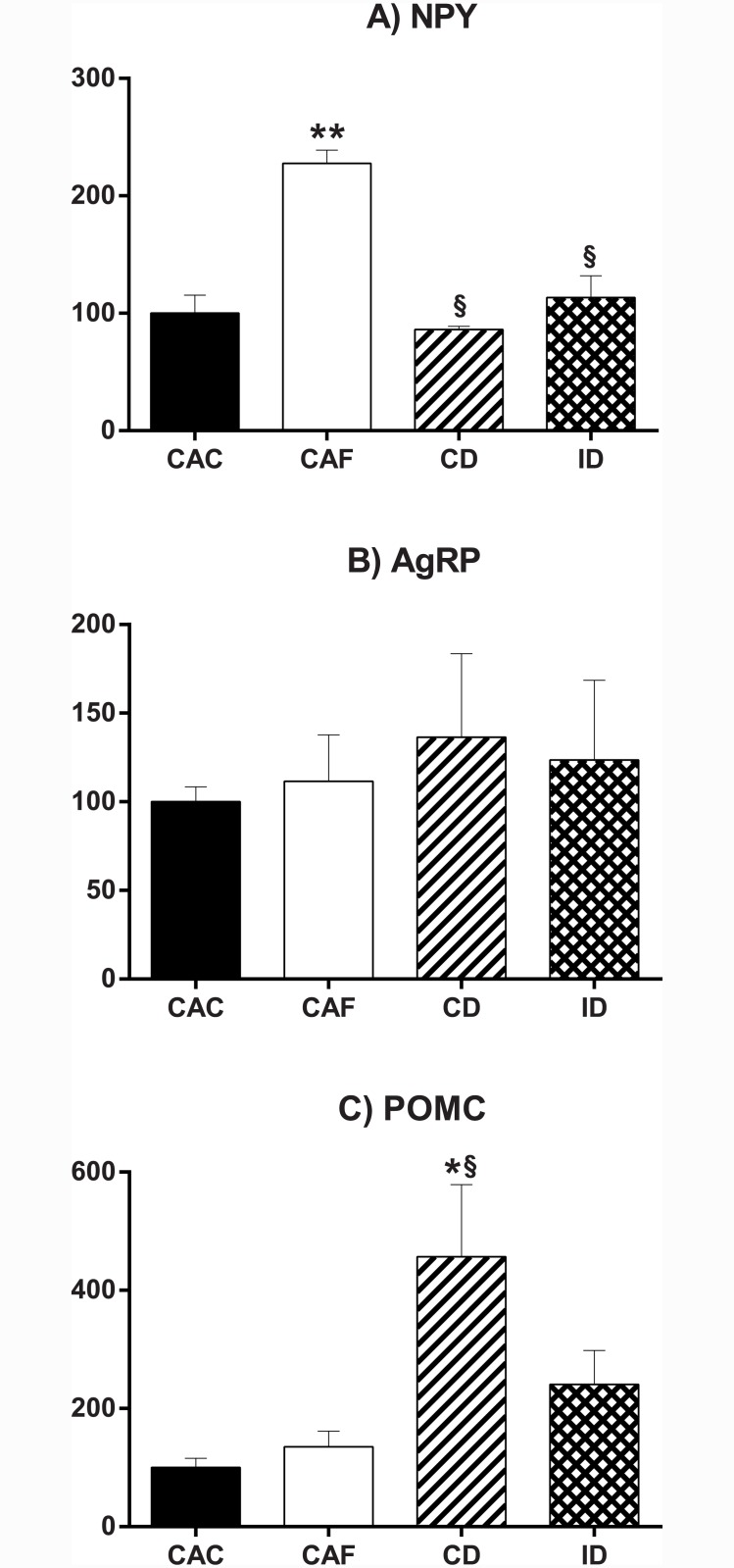
Hypothalamic expression of regulators of energy balance at the end of the weight loss phase. Whole hypothalamic blocks were used for the determination of (A) neuropeptide Y (NPY), (B) agouti related peptide (AgRP) and (C) proopiomelanocortin (POMC) mRNA expression levels by quantitative PCR. Values are expressed as fold-change in expression relative to that of the housekeeping gene, ribosomal protein L19 and as a percentage of CAC mice (open bars). CAC, continuous *ad libitum* chow; CAF, continuous *ad libitum* high fat diet; CD, continuous diet; ID, intermittent diet. Data are means ± SEM of 4 mice per group. * P<0.05, ** P<0.01 versus CAC. § P<0.05 versus CAF.

To explore possible mechanisms for the significantly greater weight loss efficiency with intermittent compared to continuous moderate energy restriction, we investigated hypothalamic mRNA expression of key regulators of energy homeostasis. Genes under investigation were the orexigenic peptides, neuropeptide Y (NPY) and agouti related peptide (AgRP), as well as proopiomelanocortin (POMC), which produces several peptides including the anorexigenic alpha melanocyte stimulating hormone (α-MSH) and the orexigenic opioid peptide, β-endorphin [37]. As shown in Fig 5A, whereas mice in the CAF group showed significant up-regulation of hypothalamic NPY expression relative to mice in the CAC condition, in keeping with previously observed effects of dietary obesity in rodents [38,39], and whereas hypothalamic NPY expression was reduced relative to values in CAF mice in both groups of energy-restricted mice, there was no significant difference in this parameter for mice on the continuous or intermittent diet. There were no significant differences in hypothalamic AgRP mRNA expression amongst mice in any of the four groups (Fig 5B). In contrast, mice on the continuous diet showed a dramatic and significant increase in hypothalamic POMC mRNA expression levels relative to control mice (CAC, CAF), and no such significant effect was seen in mice on the intermittent diet (Fig 5C).

## Discussion

This study shows that moderate energy restriction (18%), applied in intermittent bursts of 5–6 consecutive days per week and separated by 1–3 consecutive days of *ad libitum* energy intake, resulted in more efficient weight loss than continuous moderate energy restriction in diet-induced obese mice. Weight loss efficiency is defined here as the amount of weight lost per unit of energy restriction (g/kJ). So, despite eating significantly more than their counterparts on the continuous diet, mice on the intermittent diet achieved the same results in terms of reductions in body weight. Mice on the intermittent diet also achieved similar results to mice on the continuous diet in terms of reductions in body fat, lean mass, and fasting serum glucose and insulin levels, as well as a better outcome in terms of maintenance of seminal vesicle weight, an indicator of activity of the hypothalamo-pituitary-gonadal axis [40,41].

The current findings are of potential clinical significance because nutritional deficiencies, especially micronutrient deficiencies, are common amongst individuals with overweight and obesity, an effect that can be exacerbated by dietary restriction [42]. As such, achieving equivalent weight and fat loss on a higher energy intake could reduce the risk of nutritional deficiencies during weight loss interventions, provided that nutrient-rich foods are selected. Additionally, while intermittent fasting diets involving severe energy restriction appear no easier to comply with than continuous moderate energy restriction, as indicated by a lack of clear unidirectional difference in participant retention rates in clinical trials [17], a diet involving moderate energy restriction, interrupted by periods of normal food intake, may be easier to adhere to. Considering the 11.5% greater overall energy intake of mice on the intermittent versus those on the continuous diet, this difference would equate to an average of 820 kJ per day more energy intake for humans on an intermittent versus a continuous diet, assuming a weight maintenance energy requirement of 8,700 kJ per day and an energy intake of 7,130 kJ per day on a continuous diet involving 18% energy restriction. 820 kJ is the amount of energy found in a small meal or substantial snack, such as a medium-sized egg (48 g) on a thin slice of bread (30 g) spread with a teaspoon of butter or margarine (5 g) and served with a small tomato (100 g). Given that hunger is a common side effect of continuous diets involving moderate energy restriction [43–45], the possibility of losing the same amount of weight while consuming an average of 820 kJ more per day would thus likely hold appeal to people embarking on a weight loss program. This has potential implications for the long-term management of obesity, to be discussed further below.

While there is theoretical rationale to expect that intermittent energy restriction might lead to greater weight loss efficiency than continuous energy restriction, to our knowledge this is the first intermittent diet protocol to show improved weight loss efficiency relative to continuous energy restriction. Evidence that the ‘metabolic rest periods’ of *ad libitum* food intake abated the reduced energy expenditure and other metabolic changes that inhibit fat loss and increased drive to eat in our intermittent diet protocol can be seen not only from the improved weight loss efficiency, but also from the fact that whereas mice on the intermittent diet were markedly hyperphagic relative to non-dieting controls on the first day of *ad libitum* food intake, by the second and third day, energy intake was not significantly greater than that of these controls. Intermittent energy restriction has been examined in several previous publications, but relatively few studies have made head-to-head comparisons of the effects of continuous versus intermittent energy restriction on weight or fat loss in rodents [4,8–10] or humans [2,3,6,7,46–53]. While all but one [4] of these intermittent energy restriction protocols resulted in significant fat and / or weight loss compared to baseline, only one of these studies [50] showed evidence of improved weight loss or fat loss efficiency when compared to continuous energy restriction, like in the current study, as indicated by similar fat and / or weight loss despite greater reported energy intake with intermittent energy restriction. This may be at least partially because all but three [3,9,50] previous comparisons of intermittent versus continuous energy restriction used severe energy restriction in the intermittent but not in the continuous arm [2,4,6–8,10,46–49,53], and more severe energy restriction has been shown to reduce weight loss efficiency [21–23]. Of these three studies that used intermittent moderate energy restriction in mice [9] or humans [3,50], there was insufficient data available to definitively assess weight loss efficiency compared to continuous moderate energy restriction. It is notoriously difficult to control or even just assess energy intake in free-living humans [32]. Thus the current study in mice—while essentially descriptive in nature—has provided insight into the weight loss efficiency of intermittent versus continuous moderate energy restriction, insight that would be difficult to attain in free-living humans. In summary, we believe that the use of *moderate* energy restriction as part of intermittent dieting strategies is a promising angle of investigation for future human obesity treatments.

We initially hypothesized that the mechanism by which intermittent moderate energy restriction improved weight loss efficiency and maintenance of seminal vesicle weight could involve attenuation of hypothalamic expression of the orexigenic peptides, NPY and AgRP. Indeed, energy restriction in rodents increases the hypothalamic expression of NPY and AgRP, which induce a heightened drive to eat as well as a reduction in energy expenditure and inhibition of the hypothalamo-pituitary-gonadal axis, as previously reviewed [24,25]. It is also known that the effect of energy restriction to increase hypothalamic NPY or AgRP expression is significantly reduced or completely abolished within one day of *ad libitum* access to food [54–57]. Contrary to our hypothesis, however, we did not observe any effect of continuous moderate energy restriction to increase hypothalamic expression of NPY or AgRP mRNA relative to controls with continuous *ad libitum* chow access, perhaps because moderate energy restriction was used in our current experiments, unlike other publications that used severe energy restriction (fasting) [54–58]. Also contrary to our hypothesis, we did not observe any difference between mice on the continuous and intermittent diets with respect to hypothalamic NPY or AgRP concentrations. What we did see, however, was a significantly greater hypothalamic POMC mRNA expression in mice on the continuous diet versus controls with *ad libitum* chow access, with no such significant increase seen in mice on the intermittent diet. While the hypothalamic POMC gene produces the anorexigenic peptide, α-MSH, it also produces several other products with opposing effects on food intake or energy metabolism, one of which is β-endorphin [37]. β-endorphin increases energy intake and body weight [59], at least partially via antagonism of the anorexigenic and weight-reducing effect of α-MSH [60]. β-endorphin is also known to inhibit the hypothalamo-pituitary-gonadal axis in several species, via actions in the hypothalamus and peripheral tissues [61,62], consistent with the significantly reduced seminal vesicle weight seen in mice on the continuous but not the intermittent diet. These suppositions are speculative, however, and further work would be required to show whether or not reduced hypothalamic POMC expression—as well as other pathways, such as reduced gut nutrient absorption, increased thyroid function and increased energy expenditure—might be related to the effects of intermittent versus continuous energy restriction.

Despite exhibiting improved weight loss efficiency, mice on the intermittent diet were not protected from weight regain upon longer-term reinstatement of *ad libitum* access to normal chow or the high fat diet, gaining just as much weight and fat mass as animals on the continuous diet. Therefore, as is often the case in people with a body mass index in the obese range, long-term maintenance of the reduced body weight would require ongoing energy restriction. Given that energy restriction that is deliberately interrupted with periods of *ad libitum* food intake would conceivably be easier to adhere to than continuous energy restriction, we believe that this strategy may be more suitable for long-term maintenance of a reduced body weight than continuous energy restriction. Further work is required to assess these possibilities in humans.

Weight loss is known to improve glucose homeostasis and to reduce the risk of progression to type 2 diabetes mellitus [1]. Most head to head comparisons have suggested that intermittent severe energy restriction produces similar benefits to continuous moderate energy restriction for reducing fasting circulating levels of glucose, insulin and glycated haemoglobin (HbA_1c_), as recently reviewed [17]. Only three publications in humans have reported a greater decrease in fasting circulating concentrations of glucose [3,7], insulin or HOMA-IR [6,7] with certain forms of intermittent severe energy restriction versus continuous moderate energy restriction. Similarly, one study in mice showed greater reductions in fasting or glucose- or insulin- induced circulating glucose or insulin levels with intermittent compared to continuous energy restriction [8]. In contrast, another study in rats showed no effect of intermittent severe energy restriction to reduce plasma glucose concentrations, unlike the hypoglycemic effect of continuous moderate energy restriction [10]. In the current study we did not find evidence of any greater benefit of intermittent over continuous moderate energy restriction for reducing circulating glucose or insulin concentrations, or HOMA-IR, in mice, albeit animals were not studied in response to *in vivo* challenges such as glucose or insulin injection, or analysis of insulin signaling in insulin target tissues, which could have revealed subtle differences not apparent in the fasting glucose and insulin concentrations assessed here. Further, dedicated studies with a larger number of mice would be required to fully address this.

Moderate energy restriction is frequently used for the management of overweight and obesity in humans. The results from this study in mice raise the possibility that the efficiency of moderate energy restriction for weight loss could be significantly improved if it were applied intermittently, by allowing regular periods of *ad libitum* food intake. This finding has potential benefits for ensuring nutritional adequacy and long-term compliance with the diet. Further work is required to determine the optimum timing of such ‘metabolic rest periods’ when applied in humans. Moreover, care would need to be taken to ensure that the overall diet selected is nutrient-rich and not obesogenic, and that the level of intake is in accordance with physical hunger signals or energy requirements, not factors such as habits or emotions that could lead to excessive food intake and weight gain.

## Supporting Information

S1 ARRIVE Checklist (DOCX)

## Abbreviations

ANOVA: Analysis of variance
AUC: Area under the curve
BAT: Brown adipose tissue
CAC: Continuous *ad libitum* chow
CAF: Continuous *ad libitum* high fat diet
CD: Continuous diet
HOMA-IR: Homeostatic model assessment for insulin resistance
ID: Intermittent diet
SEM: Standard error of the mean
WAT: White adipose tissue
WATe: Epididymal white adipose tissue
WATi: Inguinal white adipose tissue
WATm: Mesenteric white adipose tissue
WATr: Retroperitoneal white adipose tissue
