# Supplementary material for: Intermittent Moderate Energy Restriction Improves Weight Loss Efficiency in Diet-Induced Obese Mice
Source: PLoS One. 2016 Jan 19;11(1):e0145157. doi: 10.1371/journal.pone.0145157 (PMC4718562; doi:10.1371/journal.pone.0145157)

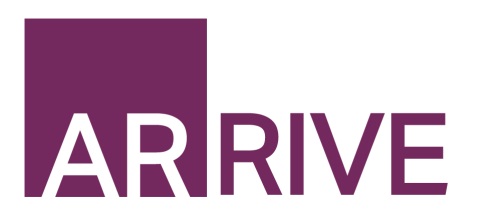


The ARRIVE Guidelines Checklist

Animal Research: Reporting In Vivo Experiments

Carol Kilkenny^1^, William J Browne^2^, Innes C Cuthill^3^, Michael Emerson^4^ and Douglas G Altman^5^

*^1^The National Centre for the Replacement, Refinement and Reduction of Animals in Research, London, UK, ^2^School of Veterinary Science, University of Bristol, Bristol, UK, ^3^School of Biological Sciences, University of Bristol, Bristol, UK, ^4^National Heart and Lung Institute, Imperial College London, UK, ^5^Centre for Statistics in Medicine, University of Oxford, Oxford, UK.*

|  | | ITEM | RECOMMENDATION | Section/ Paragraph |
| --- | --- | --- | --- | --- |
| 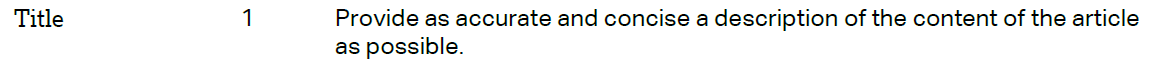 | | | Title |  |
| 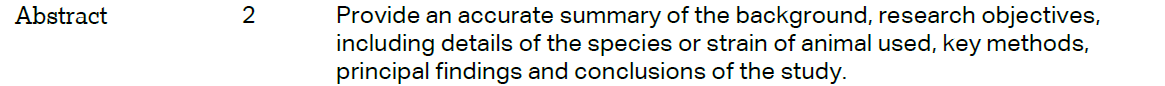 | | | Abstract |  |
| INTRODUCTION | | |  |  |
| 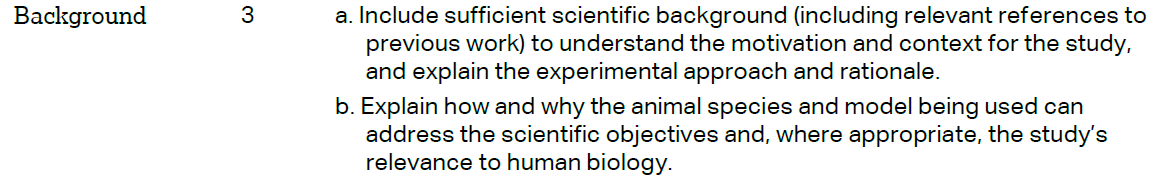 | | | Paragraphs 1-4  Paragraph 4 |  |
| 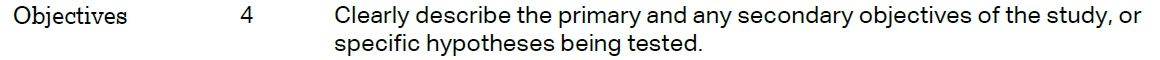 | | | Paragraph 4 |  |
| METHODS | | |  |  |
| 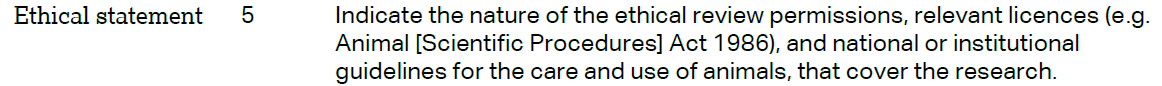 | | | Paragraph 1 |  |
| 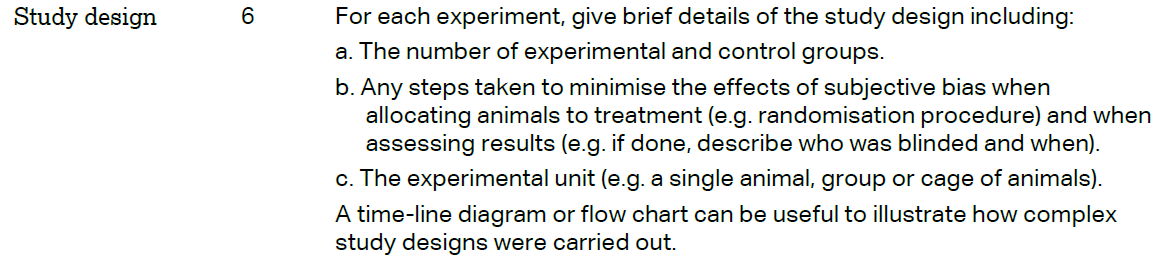 | | | Paragraph 2-3  Paragraph 3  Paragraph 2  Figure 1 |  |
| 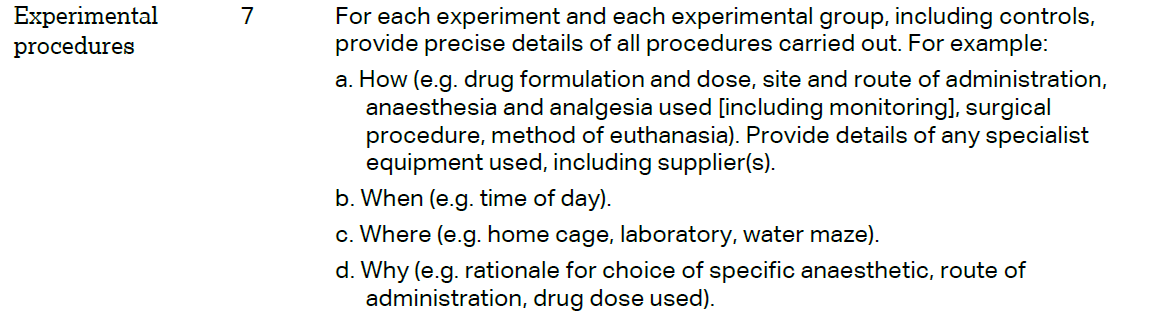 | | | Paragraphs 2-3 and 5-6 |  |
| 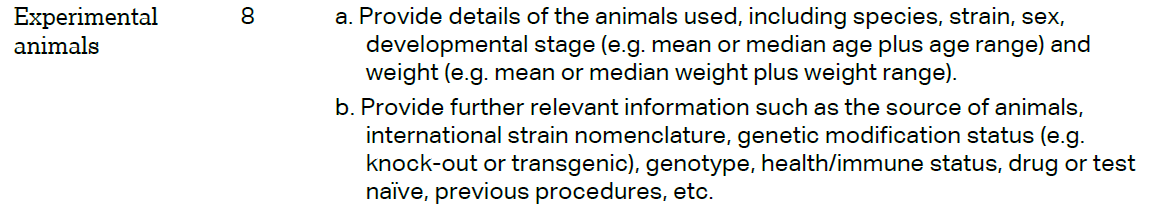 | | | Paragraph 2 |  |

The ARRIVE guidelines. Originally published in *PLoS Biology*, June 2010^1^

| 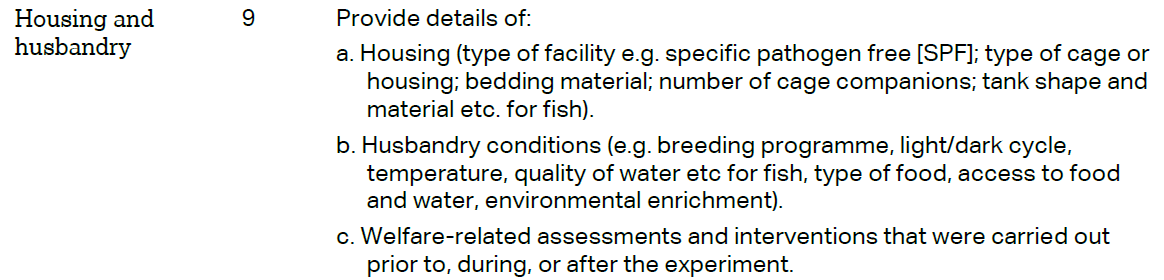 | Paragraph 2 | |
| --- | --- | --- |
| 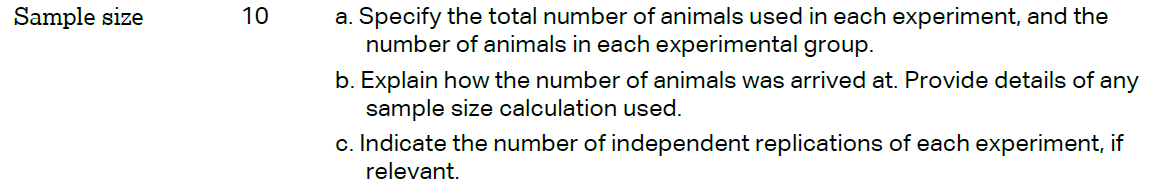 | Paragraphs 2, 3 and 5 | |
| 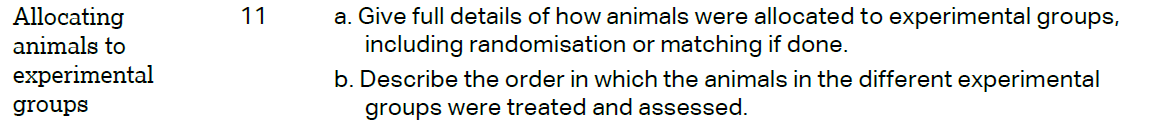 | Paragraphs 2-3 and 5-6 | |
| 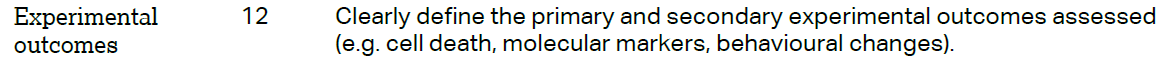 | Paragraphs 3, 5, 7, 8 | |
| 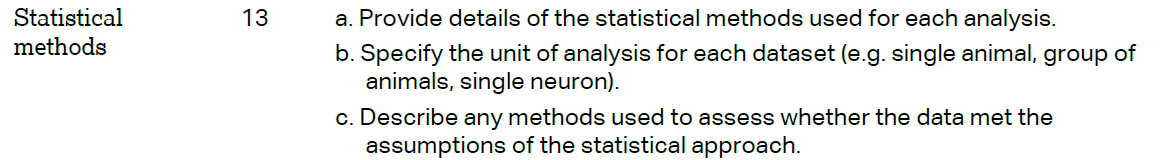 | Paragraph 9  Paragraphs 2-3 and 4 | |
| RESULTS |  | |
| 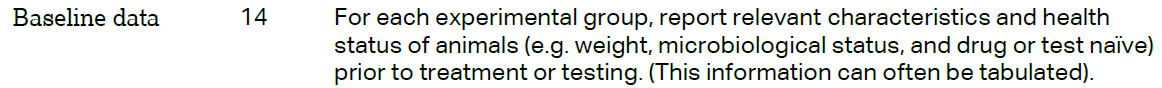 | Methods Paragraph 2 | |
| 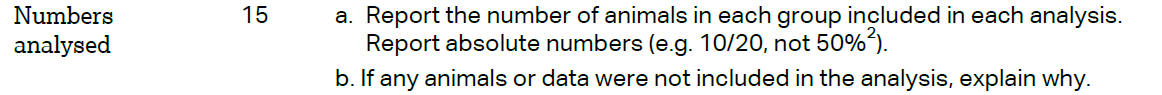 | Methods Paragraphs 2-5  Methods Paragraph 3 | |
| 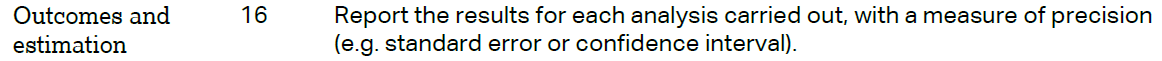 | Figures 2-5  Tables 1-2 | |
| 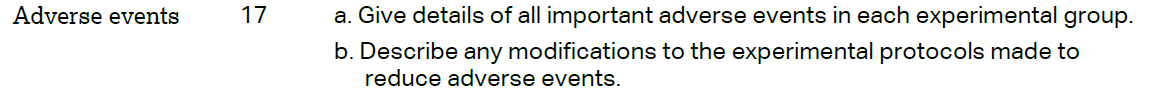 | - | |
| DISCUSSION |  | |
| 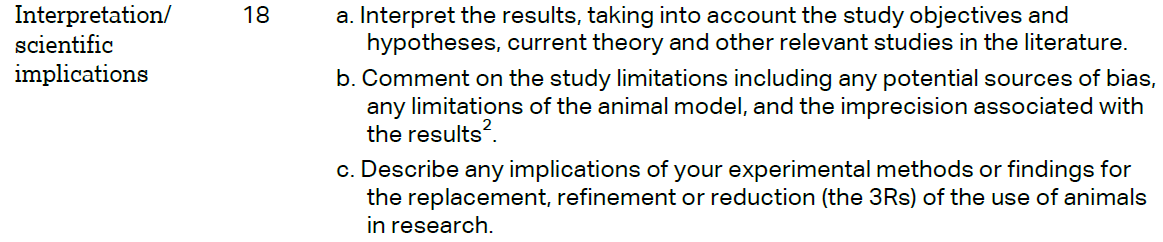 | Throughout | |
| 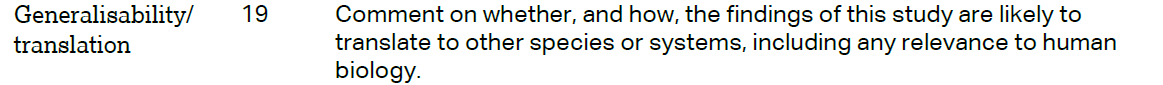 | DiscusssionParagraph 7 | |
| 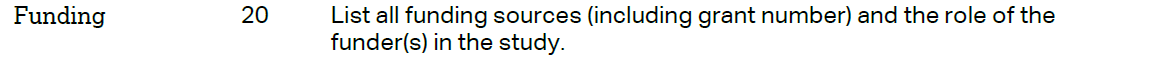 | | Acknowledgements |


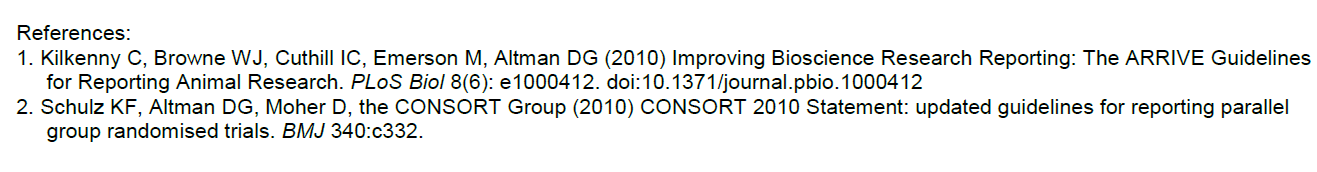

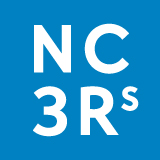

Supplement: S1 ARRIVE Checklist — (DOCX) [file pone.0145157.s001.docx]
